# Effects of *Lactococcus lactis* Strain Plasma (LC-Plasma) Intake on Infection-Related Symptoms Among Healthcare Workers: A Randomized, Double-Blind, Placebo-Controlled Study

**DOI:** 10.3390/tropicalmed11050121

**Published:** 2026-05-05

**Authors:** Zhao Xuan Low, Nghiem Nguyet Thu, Truong Tuyet Mai, Tran Thanh Duong, Pouya Hassandarvish, Vunjia Tiong, Nguyen Thi Thu Thuy, Nguyen Thi Tham, Cap Minh Duc, Osamu Kanauchi, Sazaly Abubakar

**Affiliations:** 1 Tropical Infectious Disease Research and Education Centre (TIDREC), Universiti Malaya, Kuala Lumpur 50603, Malaysiakanauchio@kirin.co.jp (O.K.); 2 National Institute of Nutrition, 48B Tang Bat Ho, Pham Dinh Ho, Hai Ba Trung, Hanoi 100000, Vietnam; 3 Department of Biomedical Science, Faculty of Medicine, Universiti Malaya, Kuala Lumpur 50603, Malaysia; 4 72A Nguyen Binh Khiem, Hai Phong 100000, Vietnam; nttham@hpmu.edu.vn (N.T.T.);; 5 Institute of Health Sciences, Kirin Holdings Co., Ltd., 2-26-1, Muraoka-Higashi, Fujisawa 251-8555, Kanagawa, Japan

**Keywords:** *Lactococcus lactis*, probiotics, antiviral, immunity, plasmacytoid, dengue, healthcare workers

## Abstract

The rising health threat to healthcare workers (HCWs) demands innovative preventive solutions that are affordable, scalable, and easy to deploy, especially in resource-limited settings. This present study investigated the effects of *Lactococcus lactis* strain Plasma (LC-Plasma) intake on upper respiratory infection (URI)-like symptoms in a healthy healthcare-associated population in Vietnam. A randomized, placebo-controlled, double-blind, parallel-group clinical trial was conducted, integrating clinical symptom analysis with ex vivo immune response analysis of peripheral blood mononuclear cells (PBMCs). The study found that after 4 weeks of continuous oral LC-Plasma intake, participants in the LC-Plasma group had significantly fewer cumulative days of fever and fatigue than those in the Control group. Increased expression of interferon-stimulated genes (ISGs), particularly MxA, was observed in PBMC cultures from the LC-Plasma intake group. In PBMCs from LC-Plasma recipients classified as low IFN-α responders, the addition of CpG ODN 2216, a mild TLR9 agonist, significantly enhanced interferon-α production. Humoral factors derived from LC-Plasma-primed PBMCs demonstrated inhibitory effects on dengue virus replication in Huh-7 cells. These results suggest that LC-Plasma consumption by the healthcare-associated population reduces the severity of viral infection symptoms, notably fever and fatigue. Elevation of systemic antiviral immunity through activation of plasmacytoid dendritic cells (pDCs) to produce IFN-α and upregulation of ISG expression could be the mechanisms of action. *Lactococcus lactis* LC-Plasma supplementation, hence, presents a promising adjunctive approach to alleviate the burden of URI-like symptoms in low-resourced vulnerable populations.

## 1. Introduction

Frontline healthcare workers (HCWs) face disproportionately high exposure to infectious diseases due to the nature of their work and their direct, frequent contact with patients [1]. Past outbreaks, including SARS, MERS, Ebola, and COVID-19, demonstrated how emerging pathogens can cause severe illness and elevated mortality among HCWs. Although such high-impact emerging infections are of major concern, they tend to occur sporadically or in limited geographic regions. In contrast, HCWs are daily exposed to far more common pathogens, such as rhinoviruses, enteroviruses, adenoviruses, and a range of bacterial infections. Occupational exposure studies highlight this persistent risk. For example, a study in Vietnam reported that 29.1% of 55 HCWs carried methicillin-resistant *Staphylococcus aureus* (MRSA) on their hands or in their nasal passages, with 24.5% identified as persistent nasal carriers; nursing assistants had the highest carriage rates, underscoring the occupational hazards faced by HCWs [2,3]. In dengue-endemic regions such as Vietnam, HCWs have also been shown to be at increased risk of nosocomial dengue infection, not only through mosquito-borne transmission but also via atypical routes including mucocutaneous exposure, needlestick injuries, and possible aerosol transmission [4,5,6,7,8]. To reduce their risk of infection, HCWs must consistently use personal protective equipment (PPE) such as masks, gowns, gloves, face shields, and respirators, and are often required or strongly encouraged to receive available vaccines, particularly for respiratory infections like influenza and COVID-19. While these measures are effective, they impose significant financial and logistical burdens on healthcare systems, especially in low-resource settings, leading to inconsistent implementation and leaving HCWs vulnerable. These challenges highlight the need for affordable, scalable, and easy-to-deploy, adjunctive, innovative, and preventive strategies that HCWs can adopt to strengthen their immunity and reduce the severity of infections when exposure occurs.

Recently, the role of probiotics in the prevention and treatment of infectious diseases has become a significant focus of research. Studies have shown that several probiotics effectively prevent or treat virally induced infectious diseases [9]. For example, a meta-analysis suggested that probiotics could serve as an adjunct treatment to reduce mortality risk or improve clinical outcomes in COVID-19 patients [10]. Probiotics, which produce lactic acid as a byproduct, are classified as lactic acid bacteria (LAB), a diverse group of rod- or cocci-shaped Gram-positive bacteria widely used to produce food and supplements with probiotic properties. Ingestion of LAB can aid in lactose digestion, help prevent and treat diarrheal diseases, and enhance the immune function of the digestive tract [11,12]. Given their demonstrated health benefits, there is growing interest in further developing functional foods enriched with probiotics to enhance their infection-preventing properties. One example is *Lactococcus lactis* strain Plasma (LC-Plasma), also called *Lactococcus lactis* subsp. *lactis* JCM5805. It is a unique LAB strain incorporated into functional foods such as yoghurt beverages. LC-Plasma has been reported to improve gastrointestinal health, alleviate flu-like symptoms, and enhance overall quality of life.

Notably, LC-Plasma can activate pDCs or PBMCs to increase the production of type I and III interferons (IFNs), leading to broad-spectrum antiviral effects in vitro [13,14]. In a murine parainfluenza virus type 1 (mPIV1) model, LC-Plasma intake upregulated antiviral gene expression and decreased lung inflammation compared to the controls, suggesting a protective effect against mPIV1 infection. Similarly, in a DENV model, two-week oral LC-Plasma treatment reduced viral titers and downregulated the expression of inflammatory markers, including IL-6, MCP-1, and IFN-γ in splenocytes, and TNF-α in hepatic cells. These data indicate that LC-Plasma may modulate host immune responses, thereby attenuating viral pathogenesis and associated inflammation [13,14,15,16,17].

Previous clinical trials of LC-Plasma have suggested that oral administration prevents the onset of influenza-like illnesses in healthy Japanese adults by upregulating IFN-α-mediated responses [18] and significantly attenuates dengue fever-like symptoms in healthy Malaysian adults [19]. LC-Plasma was also found to reduce the cumulative number of school absence days due to infectious diseases and to alleviate symptoms among elementary school children in Vietnam [20]. However, these clinical trials primarily focus on individual daily symptom scores or clinical endpoints, which are subjective, limiting their ability to provide specific insights into immune cell functions or elucidate the immunomodulatory mechanism of LC-Plasma.

This study is a randomized, double-blind, placebo-controlled, parallel-group clinical trial conducted among healthy medical student volunteers in Vietnam during their clinical placements. These students were considered an appropriate surrogate population for frontline healthcare workers within the participating healthcare facilities. The trial integrates clinical symptom scoring with peripheral blood mononuclear cell (PBMC) functional assays to explore the potential immune mechanisms activated by oral LC-Plasma intake. The primary objective is to determine whether LC-Plasma consumption reduces the severity of upper respiratory infection (URI)-like symptoms in individuals functioning in healthcare settings.

## 2. Materials and Methods

### 2.1. Statement of Ethics

The study design was approved by the Institutional Review Board (IRB) of the National Institute of Nutrition (NIN) (Approval No. 1314/VDD-QLKH) and the NIN Scientific Committee (Approval No. 1621/QD-VDD). All procedures involving human participants adhered to the ethical standards set forth by the institutional and/or national research committees and conformed to the principles outlined in the 1964 Helsinki Declaration and its subsequent amendments or equivalent ethical standards. This study is registered with the University Hospital Medical Information Network Clinical Trial Registry (Registration No. UMIN 000053690) (Registration Date: 22 February 2024) (https://center6.umin.ac.jp/cgi-open-bin/ctr/ctr_view.cgi?recptno=R000061269 accessed 1 November 2021). Written informed consent was obtained from all study participants. Although trial registration was slightly delayed due to procedural requirements, participant recruitment commenced immediately after IRB approval. Participant selection, baseline (pre-intervention) assessments, and the intervention were carried out prospectively. The study received full IRB approval in Vietnam prior to the commencement of participant recruitment. Following receipt of this approval, recruitment was initiated in accordance with the approved protocol. Due to administrative oversight during this period, the clinical trial registration was completed after recruitment had begun.

### 2.2. Study Design

**Figure 1:**
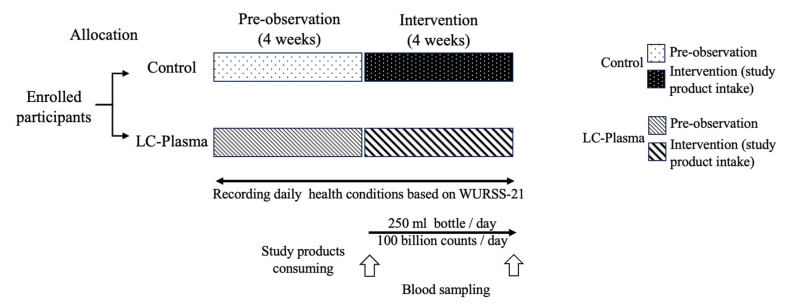
This figure shows the timeline and brief design of this clinical trial. After the allocation, participants consumed one bottle of the study product for 4 consecutive weeks. From the pre-observation period, daily health conditions were recorded until the end of the intervention period. Whole blood was collected before (0 week) and after (4th week) the intervention period.

The study took place at the Hai Phong University of Medicine and Pharmacy in Hai Phong, Vietnam, from 30 November 2023 to December 2024. Hai Phong University is a medical school where students undergo clinical training, which puts them at high risk of infection. This study was a randomized, placebo-controlled, double-blind, parallel-group study conducted by the NIN in Vietnam. During the pre-intervention period, which lasted 4 weeks, participants recorded their daily health conditions in the provided record booklet, and their basic health status was closely monitored (Figure 1). In November 2023, 110 healthy Vietnamese students in clinical training at Hai Phong University of Medicine and Pharmacy were recruited according to predefined inclusion and exclusion criteria. We enrolled medical students rather than nurses or physicians, because recruiting sufficient numbers from those groups was impractical and because blood sampling and monitoring could disrupt hospital workflows. Medical students in clinical training have frequent exposure to patients with infectious diseases and are therefore considered to be at higher risk than the general population. Sample size estimation was based on findings from a previous study, assuming an alpha error probability of 0.05, a power (1-β) of 0.8, and an effect size of 0.5 [21]. Based on the G*Power analysis 3.1.9.7 (http://www.gpower.hhu.de/ 1 November 2021), the estimated sample size was approximately 50 participants per group. To account for a dropout rate of approximately 10%, the final sample size was adjusted to 55 participants per group, for a total of 110 participants. After enrolment, participants were randomly assigned in a 1:1 ratio to either the active (LC-Plasma) group (*n* = 55) or the Control group (*n* = 55). The random allocation sequence was conducted in the order of participant admission using Microsoft Excel and the RAND function. Allocation concealment was ensured using sequentially numbered, opaque, sealed envelopes prepared by an independent study group allocator. To minimize bias, participants, investigators, care providers, and outcome assessors were blinded to group allocation throughout the study.

**Figure 2:**
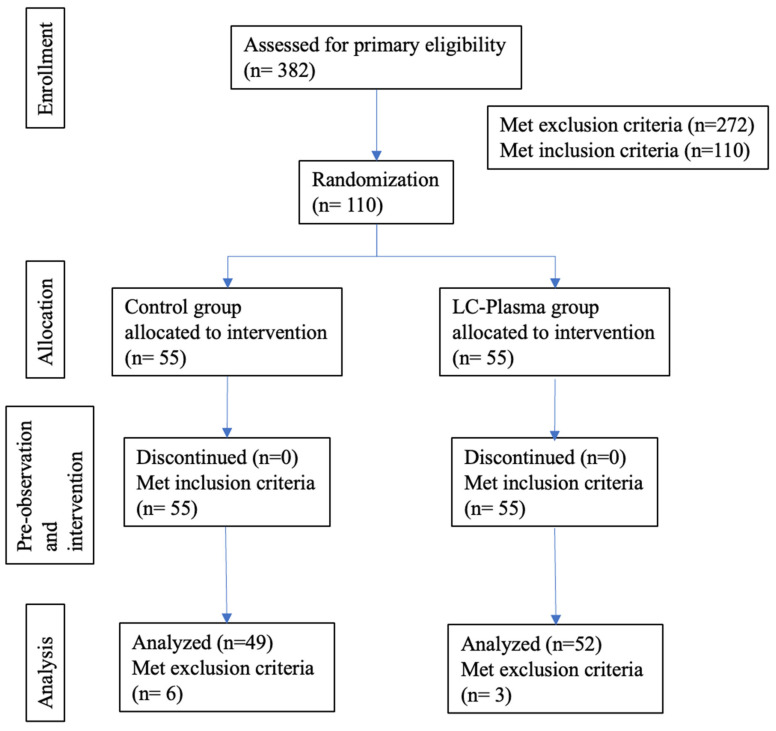
Flow chart diagram of this trial.

After the allocation, the participants in the LC-Plasma group consumed one bottle of the LC-Plasma beverage product daily for four consecutive weeks (intervention period from 2 January 2024 to 29 January 2024), as recorded in a record booklet and by counting the empty bottles. In comparison, those in the Control group consumed one bottle of the placebo product daily. Participants recorded their daily health conditions and product intake in their record booklets throughout the intervention period. Physical examinations and blood sampling (8 mL per participant) were performed before and after the intervention period. No interim analyses for efficacy or futility were conducted. During the intervention, no participants dropped out of the study. However, three participants from the LC-Plasma group and six participants from the Control group were excluded due to non-compliance with inclusion criterion 6. As a result, the final number of participants included in the analysis was 52 in the LC-Plasma group and 49 in the Control group (Figure 2). This study was conducted in accordance with the CONSORT (Consolidated Standards of Reporting Trials) 2010 guidelines to ensure transparent and comprehensive reporting of randomized controlled trials [22].

### 2.3. Inclusion Criteria

(1) Signed and dated written informed consent form provided, after the willingness of participants was confirmed verbally.

(2) Willingness to comply with all study procedures for the period of the study.

(3) Healthy School grade 4–5, (20 < Age < 30) students with Vietnamese nationality in Hai Phong University of Medicine and Pharmacy.

(4) Persons who have received the COVID-19 vaccine at least two times and more than 2 weeks have passed after the last vaccination.

(5) Persons who are not currently infected with SARS-CoV-2, and if they were infected, at least 4 weeks have passed since recovery.

(6) No underlying acute and/or chronic sickness from respiratory disease and/or gastrointestinal disease at enrollment (pre-observation period).

### 2.4. Exclusion Criteria

(1) Unable to provide a signed and dated informed consent form.

(2) Unable to comply with all the study procedures.

(3) Allergic to milk and/or soy (contained within the study products).

(4) Regularly using steroid drugs (immunosuppressive drugs).

(5) Any persons deemed inappropriate as participants by the principal investigators of this study.

(6) Those currently participating in other clinical trials (participants) or those who are planning to participate in other clinical trials during the study period.

(7) Women who are pregnant, who intend to become pregnant during the study period, or breastfeeding mothers.

(8) Those who are unable to stop drinking alcohol for two days before sampling blood.

### 2.5. Study Product

LC-Plasma has previously been shown to activate pDCs and stimulate immune responses against the inactivated influenza virus in PBMCs at a daily dose of approximately 1.0 × 10^11^ cells per person in viable and heat-killed forms [21]. Based on this, this study’s daily dose of heat-killed LC-Plasma was set at 1.0 × 10^11^ cells per serving. The PET-bottled beverages (280 mL per serving) were formulated to contain sugar (5%), sweetener (0.01%), citric acid (0.04%), flavours (0.14%), skim milk powder (0.1%), and either 50 mg of heat-killed LC-Plasma (containing more than 1.0 × 10^11^ cells, serving as the active product for the LC-Plasma group) or 50 mg of maltodextrin (serving as the placebo product for the Control group). These beverages were manufactured under the ISO 22000 and HACCP [23] -certified safety standards by Interfood Shareholding Company in Vietnam (Ho Chi Minh City, Vietnam). The LC-Plasma and placebo products were matched for taste, colour, size, and nutritional composition, including 22.4 kcal, 0.0 g protein, 0.0 g fats, and 5.6 g carbohydrates per 100 mL, to ensure comparability between the two groups. The study products (PET-bottled beverages) are fully covered with indistinguishable white shrink film.

### 2.6. Study Outcomes

The primary endpoint of this study was to evaluate the antiviral effects of LC-Plasma using humoral factors derived from LC-Plasma- or placebo-primed PBMCs against the Dengue virus. This was assessed by stimulating LC-Plasma/placebo primed PBMCs with mild CpG DNA, followed by quantitative reverse transcription polymerase chain reaction (qRT-PCR) to quantify DENV replication. The secondary endpoints included the assessment of ISG expression (IFITM-1, ISG15, ISG20, Mx-A, OAS-1, RSAD2 (Viperin), and RyDEN) in LC-Plasma/placebo primed PBMCs. Additionally, clinical parameters were evaluated, including the Wisconsin Upper Respiratory Symptom Survey (WURSS-21) score to measure the severity of upper respiratory tract symptoms. The study also examined interferon-alpha (IFN-α) production in the plasma of LC-Plasma- or placebo-primed PBMCs as a marker of immune activation. The exploratory endpoints included assessments of general well-being using the Wisconsin Score and gastrointestinal distress (GID) symptoms, including abdominal pain, diarrhea, and vomiting severity. The frequency of bowel movements was also recorded daily.

### 2.7. Clinical Data Correction

During both the pre-intervention and intervention periods, participants documented their medical history, including any chronic diseases, acute illnesses, or other past medical conditions (categorized as positive or negative). They also recorded the use of steroids or other medications and monitored key health parameters. These included body temperature (categorized as 0 for <37.5 °C and 1 for ≥37.5 °C), URI-like symptoms using a modified self-reported WURSS-21 score (Table 1), general well-being, and gastrointestinal symptoms [20].

### 2.8. Cells and Virus

Huh-7 cells (accession number JCRB0403, JCRB Cell Bank, Tokyo, Japan) were cultured and maintained in low-glucose (1 g/L) DMEM (MT-10-009-CV, Corning, Corning, NY, USA) supplemented with 10% heat-inactivated FBS and incubated at 37 °C and 5% CO_2_. The FBS was reduced to 2% for the infection assays. The DENV-2 (New Guinea C (NGC) strain, accession number: ATCC VR-1584) was propagated in Vero cells (CCL-81, purchased from the European Collection of Animal Cell Cultures (ECACC) repository) and titrated using the foci assay [13,24].

### 2.9. Plasma and PBMC Isolation, Preservation, and Culture

PBMCs and plasma were isolated from 8 mL of whole blood collected per participant using the BD Vacutainer^®^ CPT™ Mononuclear Cell Preparation Tube (362760, BD Biosciences, Franklin Lakes, NJ, USA) according to the manufacturer’s protocol. The isolated PBMCs were cryopreserved in Cellbanker 2 (11914, AMSBIO, Tokyo, Japan) and stored at −80 °C for later analysis. Upon thawing, PBMC viability consistently exceeded 80% [13,25].

### 2.10. IFN-α Production in PBMC Culture Supernatant and Plasma

To assess IFN-α production by PBMCs, 2 × 10^5^ cells/mL were seeded into each well of a 96-well plate and stimulated with 0.05 µM of CpG ODN 2216 (tlrl-2216, InvivoGen, San Diego, CA, USA). After 24 h of incubation, the supernatant was collected by centrifugation. The IFN-α concentration in the PBMC supernatant and plasma was quantified using a human IFN-α ELISA kit (E-EL-H6125, Elabscience, Wuhan, China) according to the manufacturer’s instructions.

### 2.11. Antiviral Assay

To evaluate the antiviral effect of LC-Plasma, the PBMC supernatant collected from each participant was used to treat Huh-7 cells for 24 h before DENV-2 infection. After stimulation, Huh-7 cells were infected with infectious virus particles at a multiplicity of infection (MOI) of 0.1 for one hour at 37°C. The viral progeny in the supernatant was harvested 48 h post-infection and stored at −80°C. Viral RNA was extracted using the QIAampR Viral RNA extraction kit (QIAG-52904, QIAGEN, Hilden, Germany), following the manufacturer’s protocol. The extracted viral RNA was reverse-transcribed and amplified using a Step-OnePlus Real-Time PCR System with the SensiFast SYBR Hi-ROX one-step kit (BIO-73005, Bioline, London, UK) according to the manufacturer’s protocol. The primers were specific for the DENV capsid gene (forward: CAATATGCTGAAACGCGAGAGAAA, reverse: AAGACATTGATGGCTTTTGA) [13].

### 2.12. Interferon-Stimulated Genes (ISGs) Expression Analysis

The isolated PBMCs were analyzed for ISG expression. Total RNA was extracted from the PBMCs using the RNeasy Kit (QIAG-74106, Qiagen, Hilden, Germany), following the manufacturer’s instructions. Complementary DNA (cDNA) was synthesized using the Quantabio cDNA synthesis kit (95047-500, Quantabio, Beverly, MA, USA) following the manufacturer’s protocol. Quantitative PCR (qPCR) was performed using the TaqMan™ Fast Advanced Master Mix (4444556, Applied Biosystems, Thermo Fisher Scientific, Waltham, MA, USA) and TaqMan Array Plates (4413266, Applied Biosystems, Thermo Fisher Scientific, Waltham, MA, USA), preloaded with customized primers and probes specific to the ISGs [26]. The targeted ISGs were: RyDEN (Hs01032873_g1), IFITM-1 (Hs00705137_s1), OAS-1 (Hs00973635_m1), ISG15 (Hs01921425_s1), ISG20 (Hs00158122_m1), Viperin/RSAD2 (Hs00369813_m1), and MxA (Hs00895608_m1). The relative gene expression levels of the ISGs were calculated using the 2−ΔΔCt method [27], comparing the Ct values of ISGs before and after the intervention. Expression levels were normalized to the reference gene 18S rRNA (Hs99999901_s1).

### 2.13. Statistical Analysis

For statistical analysis, the body weight and height were compared between groups using Student's *t*-test. The Mann–Whitney test was applied to nonparametric data, such as URI, gastrointestinal, and general well-being scores. Gender distribution, cumulative days with positive fever status, positive gastrointestinal scores, and feelings of tiredness were analyzed using the Chi-square test. Figures are expressed as mean ± standard deviation (SD) and were analyzed using nonparametric tests, including the Mann–Whitney test for comparisons across groups, and the Kruskal–Wallis, including the Steel–Dwass test for subgroup analysis. All statistical analyses were performed using EZR software version 1.68 [28]. A *p*-value < 0.05 was considered statistically significant, while *p*-values < 0.1 indicated a tendency toward significance.

## 3. Results

### 3.1. Participant Characteristics and Background Data Analysis

A total of 110 medical school student volunteers were enrolled based on the participant inclusion and exclusion criteria. All participants provided written informed consent and received the study products. The participants’ physical condition was carefully recorded during the pre-intervention and intervention period. All participants completed the 4-week intervention, except for three participants in the LC-Plasma and six participants in the Control group. They were not included in the analysis due to recurrence of allergy disease (n = 1), acute infectious disease and/or high fever (n = 5), chest pain and cough (more than 3 weeks) (n = 1), rush, muscle and joint pain (n = 1), continuous cough (more than 2 weeks) (n = 1) in the pre-observation period (Figure 2). The baseline characteristics of the participants, such as body weight and height, are shown in Table 2. Body weight, height, and age did not show statistical differences between groups.

Body weight and height did not differ significantly between the groups (Student’s *t*-test). Gender did not show significant differences between the groups (Chi-square test). Data represent mean ± standard deviation.

### 3.2. URI Symptoms Analysis

The daily intensity of URI symptoms, based on self-reported WURSS-21 scores, is shown in Table 3. No significant differences were observed between the LC-Plasma and Control groups for any symptom or total score. However, the LC-Plasma group had significantly fewer cumulative fever-positive days (Table 4). Cumulative feeling-tired days during the intervention period were shown, with the LC-Plasma group showing significantly lower numbers than the Control group (Table 5). For other cumulative symptom-positive days in URIs, no significant differences were noted between the groups.

There were no significant differences between groups (Mann–Whitney test). Data represent mean ± standard deviation.

A significant difference in cumulative fever days during the intervention period was observed between the groups, as determined by the Chi-square test. The *p*-value, odds ratio, and 95% confidence interval (CI) are shown.

A significant difference in cumulative feeling-tired days during the intervention period was observed between the groups, as determined by the Chi-square test. The *p*-value, odds ratio, and 95% confidence interval (CI) are shown.

### 3.3. Gastrointestinal Score and General Well-Being Score Analysis

The daily intensity of gastrointestinal infectious disease-related symptoms (diarrhea and abdominal pain scores) and the general well-being score are presented in Table 6. While the general well-being score of the LC-Plasma group was slightly lower than that of the Control group, no significant differences were observed between the groups. There were no significant differences between groups in bowel movement.

There were no significant differences between groups (Mann–Whitney test). Data represents mean ± standard deviations.

### 3.4. IFN-a Concentration of Plasma and Producing Potency by PBMCs

**Figure 3:**
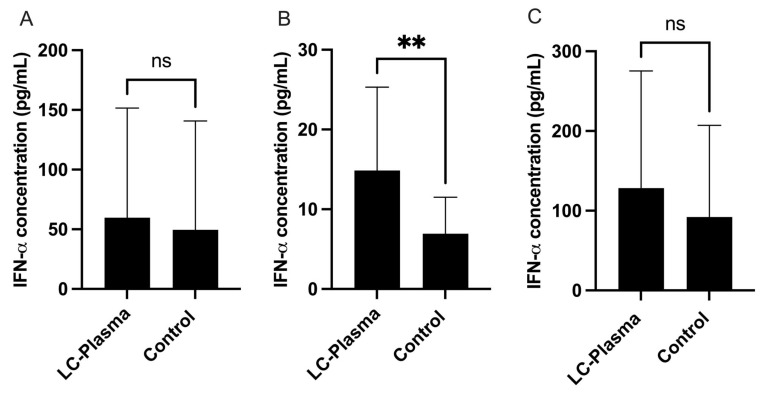
IFN-α levels in supernatants of CpG-stimulated PBMCs derived from all participants (**A**) and participants categorized as low-IFN-α responders (**B**) and high-IFN-α responders (**C**) in LC-Plasma and Control groups. IFN-α levels in the culture supernatant of CpG ODN 2216-stimulated PBMCs between low-pDC responders in the LC-Plasma group and the Control group only showed a significant difference in this ex vivo study. Bar graph presented as the mean and standard deviation (SD). Statistical significance between groups was determined using the Mann–Whitney U test; ** *p* < 0.01; ns indicates no significant (ns) difference.

At the end of the intervention period, whole blood was collected for plasma, and PBMCs were isolated. Plasma IFN-α levels showed no significant difference between the groups (LC-Plasma group: 0.75 ± 3.87 pg/mL vs. Control group: 2.29 ± 13.93 pg/mL, *p* = 0.837). Similarly, when PBMCs were stimulated with CpG ODN 2216, the IFN-α levels in the PBMC culture supernatant were higher in the LC-Plasma group (59.7 ± 91.9 pg/mL) compared to the Control group (49.5 ± 91.2 pg/mL). However, the difference was not statistically significant (*p* = 0.146) (Figure 3A). Although it was reported that direct LC-Plasma stimulation of pDCs led to a significant increase in IFN-α in vitro [14], there was no significant difference in total plasma IFN-α levels between groups in this study, despite an increasing trend in IFN-α in the LC-Plasma group following CpG stimulation of PBMCs. In light of this discrepancy between in vitro and in vivo, a more detailed mechanism study will be necessary. In our previous clinical study, the low pDC activity layer in the LC-Plasma group showed higher IFN-α transcription mRNA expression than the Control group; therefore, we attempted a stratified analysis [29]. A stratified analysis was then performed by categorizing participants into low and high pDC responders based on the median IFN-α production within each group (LC-Plasma group median: 33 pg/mL; Control group median: 15 pg/mL). The analysis revealed a significant difference in IFN-α levels in the culture supernatant of CpG ODN 2216-stimulated PBMCs between low-pDC responders in the LC-Plasma group (14.9 ± 10.5 pg/mL) and the Control group (7.3 ± 4.8 pg/mL, *p* < 0.046) (Figure 3B). However, no significant difference was observed between high-pDC responders in the LC-Plasma group (104.0 ± 112.5 pg/mL) and the Control group (96.2 ± 113.7 pg/mL) (Figure 3C). Before the intervention period, IFN-α levels in both plasma and PBMC culture supernatants did not differ significantly between groups.

### 3.5. Antiviral Replication Effects of LC-Plasma

**Figure 4:**
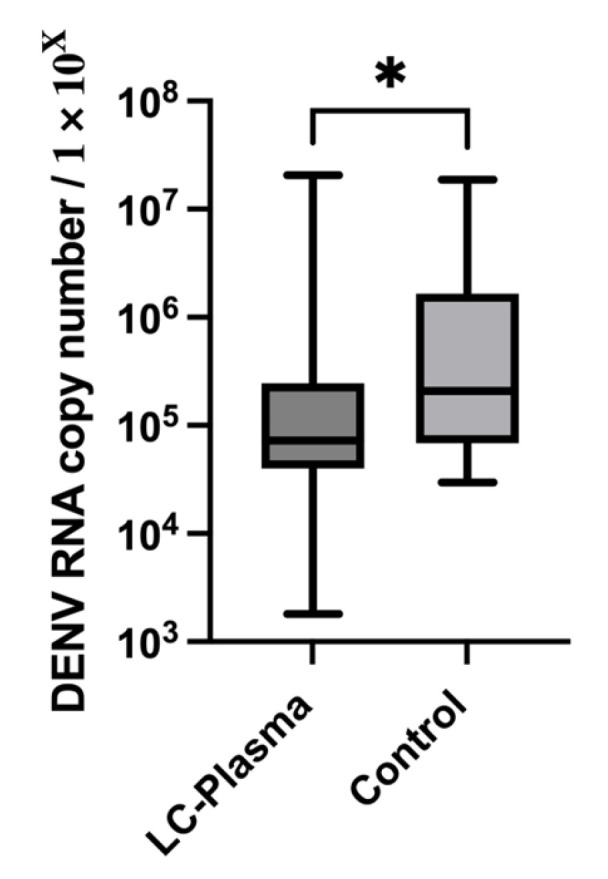
The DENV RNA copy numbers in Huh-7 cells treated with supernatants derived from CpG-stimulated PBMCs of participants in the LC-Plasma and Control groups. Box plots expressed as median ± interquartile range. The Mann–Whitney test determined statistical significance between the two groups; * *p* < 0.05.

PBMCs from both groups were stimulated with CpG ODN 2216, and the resulting humoral factors were used to evaluate the antiviral effects of oral LC-Plasma administration against DENV replication in vitro. Since we have some pre-clinical data on LC-Plasma against DENV, DENV is chosen as the representative pathogenic virus in this trial [13,19,26,30]. After the intervention period, the PBMC supernatant from the LC-Plasma group (15.9 × 10^5^ ± 38.6 × 10^5^ RNA copy number) significantly reduced (*p* = 0.013) the DENV RNA copy number compared to the Control group (16.6 × 10^5^ ± 33.0 × 10^5^ RNA copy number) (Figure 4).

### 3.6. Effects of LC-Plasma on ISG Expression in PBMCs

**Figure 5:**
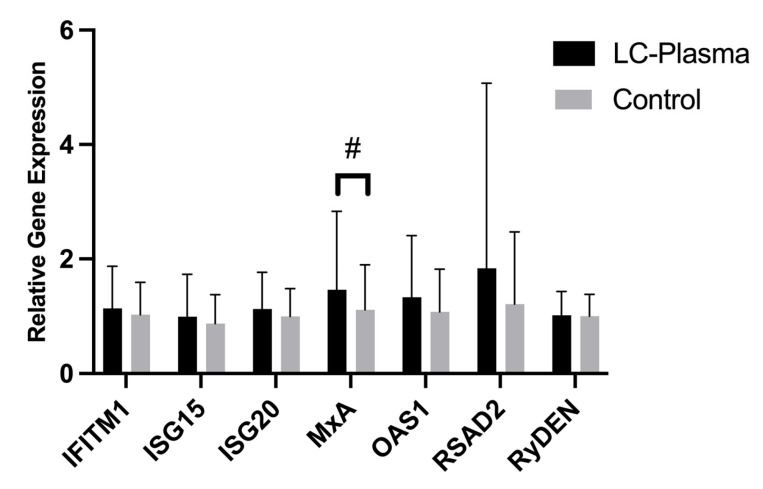
Relative ISG expression of PBMCs derived from participants in the LC-Plasma and Control groups. Data is presented as bar graphs showing the mean and SD. Statistical significance between groups was assessed using the Mann–Whitney U test. There was a trend toward a difference in MxA expression between LC-Plasma and Control (*p* = 0.0951), with #.

To assess the effects of oral LC-Plasma administration on ISG expression in PBMCs, total RNA was extracted from PBMCs in the LC-Plasma and Control groups to analyze relative ISG expression levels. After the intervention period, the LC-Plasma group demonstrated higher expression levels of OAS-1, IFITM1, ISG15, ISG20, MxA, RSAD2, and RyDEN compared to the Control group. However, only MxA showed a trend toward higher expression (*p* = 0.095) (Figure 5).

## 4. Discussion

Nutritional adequacy is vital for enhancing overall health and immunity and for preventing and mitigating infections [31], particularly among vulnerable groups such as the elderly, children, individuals with chronic metabolic conditions, and HCWs. Among these groups, the healthcare-associated population, including medical professionals, allied health professionals, medical students, and trainees, is frequently exposed to infectious agents in their work environments, significantly at risk of contracting microbial infection. A meta-analysis of 54 comparative studies highlighted that frontline HCWs face heightened risks, especially those performing high-exposure procedures such as endotracheal intubations [32]. While widely available protective measures, such as personal protective equipment and disinfectants, help reduce exposure, they do not eliminate the risk of infection. Consequently, there is a growing interest in exploring the potential of nutritional interventions to provide additional protection during viral pandemics [33]. One promising avenue is modulation of immunity through the gut microbiota, which is known to play a pivotal role in maintaining immune homeostasis and overall health [34]. Dysbiosis, or the disruption of host-microbiome balance, can increase susceptibility to infections and exacerbate diseases, impacting not only gastrointestinal health but also distant organs such as the lungs and liver [35,36]. Supplementation with probiotics has been proposed to enhance immunity in high-risk populations, including severely ill patients and frontline HCWs, potentially reducing their vulnerability to viral infections. Although robust evidence from randomized controlled trials remains limited, emerging circumstantial evidence suggests that intake of probiotics may reduce the severity of responses to infections such as COVID-19, including reducing mortality [37].

LC-Plasma, a probiotic, can be internalized by pDCs and activate pDCs to produce Type I and Type III IFNs via the TLR9-MyD88 signalling pathway [14]. The immunomodulatory effect of LC-Plasma was reported to be systemic in that IFNs produced by LC-Plasma-activated immune cells could travel to distant organs to activate liver, lung, or intestinal epithelial cells to upregulate the expression of ISGs, thus inhibiting the replication of viruses such as DENV, parainfluenza, and rotavirus, respectively [15,17,26]. Other immunomodulatory effects of LC-Plasma include alleviating DENV-mediated inflammation by reducing IL-6, MCP-1, and TNF-α, while activating NK cells via dendritic cells, thereby increasing IFNγ and granzyme production [30,38,39]. In clinical trials, LC-Plasma has been shown to activate human pDCs in PBMCs, particularly in individuals with initially low pDC activity, thus reducing common cold symptoms [18]. It has also been reported to have prophylactic effects against influenza-like illnesses, including reduced symptom-positive days, increased IFN-α expression, and upregulation of pDC activation markers [18,21]. It also reduces influenza incidence and improves immune markers, such as secretory IgA and CD86, in healthy Japanese adults [40,41], alleviates dengue fever-like symptoms in Malaysian adults [19], and reduces school absences due to infections in Vietnamese children. While these clinical studies provide valuable scientific data supporting the immunomodulatory effects of L.C-Plasma, further investigation is needed to elucidate the cellular mechanisms by which LC-Plasma exerts its effects on URI-like symptoms.

This study demonstrated that 4 weeks of LC-Plasma oral administration showed no significant difference in plasma IFN-α levels between the LC-Plasma and Control groups. To further verify this, we used a mild TLR9 agonist, CpG ODN 2216, to stimulate the PBMCs isolated from both the LC-Plasma and Control groups. However, the results again showed no significant differences between the LC-Plasma and Control groups after intervention. A previous study similarly reported that IFN-α transcriptional levels were not significantly regulated in the LC-Plasma group [41]. When participants were stratified by plasmacytoid dendritic cell (DC) activity into low and high DC responder groups, IFN-α transcriptional levels were significantly upregulated in the LC-Plasma group within the low pDC activity subgroup, but not within the high pDC activity subgroup [42]. Based on these previous findings, we performed a similar stratification analysis. We observed a significant increase in IFN-α levels in the culture supernatant of CpG ODN 2216-stimulated PBMCs from low-pDC responders in the LC-Plasma group compared to the Control group. It was previously reported that LC-Plasma increased IFN-α transcription levels in the low pDC activity group. While the previous study examined only transcriptional (gene expression) levels, our study evaluated translational (protein) levels, and both studies demonstrated a similar trend [42]. Therefore, the difference in pDC activity between the LC-Plasma and Control groups may be attributed to the greater susceptibility of participants with low pDC activity to LC-Plasma stimulation. In our previous studies restricted to infected subjects (COVID-19) [43], significant differences in IFN production and pDC activation were observed. On the other hand, in the present study, which involved healthy volunteers, IFN was measured after CpG stimulation. We also consider it plausible that stimulation by LC-Plasma is weaker than that induced by viruses and/or infectious diseases, suggesting that this food-derived material is relatively safe and does not trigger immune overstimulation.

Additionally, 4 weeks of LC-Plasma oral administration significantly upregulated ISG expression in PBMCs compared with the Control group, with MxA showing the greatest tendency to upregulate. Similar findings were reported in an ex vivo study, where humoral factors derived from LC-Plasma-stimulated PBMCs significantly increased MxA expression in human liver Huh-7 cells, resulting in broad-spectrum antiviral effects [13]. Mx proteins, including MxA, are conserved dynamin-like large GTPases with potent antiviral activity against RNA viruses. Although these reports are in vitro studies, they function by forming oligomers around viral nucleoproteins (NPs), thereby preventing viral ribonucleoproteins (vRNPs) from entering the nucleus and inhibiting viral transcription [43,44,45]. By upregulating ISGs, particularly MxA, LC-Plasma may provide protective effects against viral infections. Supporting this, another clinical trial demonstrated that oral LC-Plasma administration enhanced the immunological response to influenza A virus by increasing ISG15 transcription [18]. Since LC-Plasma upregulated ISG expression in PBMCs, we hypothesized that humoral factors derived from these PBMCs might exhibit antiviral effects upon CpG ODN stimulation. Our results showed that humoral factors from LC-Plasma-primed PBMCs reduced DENV RNA copy number compared with the Control group, indicating that LC-Plasma can stimulate humoral factors that inhibit DENV replication. This effect might be attributed to the higher IFN-α levels produced in response to oral LC-Plasma stimulation, which play a crucial role in pDC activation. We postulate that this mode of action contributed to the alleviation of dengue fever-like symptoms in Malaysian adults who consumed LC-Plasma [19].

In the clinical symptoms analysis, the cumulative days of fever and tiredness, which are related to pyrexia, were significantly lower in the LC-Plasma group than the Control group over the study period. This suggests that the intake of LC-Plasma in humans may induce a prophylactic effect against URIs, as observed in our previous clinical trial [20]. Since LC-Plasma has been shown to increase IFN-α levels and upregulate ISG expression, it might help reduce viral copies, potentially reducing the intensity and duration of symptoms such as fever and fatigue, which are often triggered by the body’s immune system in response to high viral loads. In addition, although it was difficult to show a clear reduction in symptom severity, the frequency of symptom occurrence and reproducibility have been demonstrated in previous clinical studies [19,20], and we therefore consider the present findings to be reasonable. Unlike pharmaceutical agents, LC-Plasma may not exert a strong effect on each symptom. However, the present study also supports its ability to reduce the overall frequency of unpleasant symptoms in participants. It is necessary to implement future clinical studies focusing primarily on infected subjects. LC-Plasma oral administration was reported to activate pDCs, a major producer of type I IFNs, which could help regulate the immune response [13,15,26,46] and allow for effective reduction in viral copies without overstimulating the immune system, thereby alleviating these symptoms. Additionally, the IFNs produced upon LC-Plasma stimulation might also activate other immune cells, such as natural killer [38] and cytotoxic T cells, to target and eliminate infected cells. This coordinated immune response could accelerate recovery and mitigate the systemic effects of URI-like symptoms, including pyrexia and tiredness. However, it is known that in vitro data cannot be directly linked to in vivo or clinical results. We previously reported that LC-Plasma exerts antiviral effects against upper respiratory tract infection viruses through IFN production [47], in addition to its activity against tropical viruses [13]. While it remains challenging to directly demonstrate the mechanism of action in clinical studies, these reports could support the contribution of LC-Plasma to the reduction in the number of days with virus-infection-like symptoms observed in the present study. It is considered difficult to observe a clear increase in type-1 IFN in healthy individuals who are not infected with viruses or other pathogens. IFNs are typically induced by viral infection, and therefore their circulating levels are generally low in healthy, uninfected individuals. LC-Plasma is directly taken up by pDCs and induces an increase in type-1 IFN. However, this effect is mainly observed in ex vivo experiments—mimicking direct contact between LC-Plasma and immune cells, including pDCs [48]. In this regard, future studies will be required, such as trials that enrol virus-infected participants immediately after infection diagnosis, or large-scale prospective observational studies, which are planned for future investigation. The last limitation is setting the intervention period at 4 weeks, based on a previous clinical study [49]. Although LC-Plasma demonstrated clinical efficacy by activating pDC in healthy volunteers within 2 weeks, its long-term efficacy should be evaluated in the near future across several additional points (e.g., seasonal interactions).

## 5. Conclusions

The healthcare-associated population faces heightened infection risks, emphasizing the importance of immune resilience. This study highlights that supplementation of the diet with LC-Plasma is a promising immunomodulatory agent that has been shown to increase IFN-α levels via pDC activation and to upregulate ISG expression, particularly MxA. These effects could contribute to viral inhibition, reducing viral copies and reducing symptoms of pyrexia and fatigue. However, our study has limitations, as the ex vivo design and reliance on humoral factors derived from LC-Plasma-primed PBMCs may not fully mimic in vivo conditions or directly reflect outcomes in infected individuals. Further clinical trials are needed, particularly in infected populations, to confirm LC-Plasma’s efficacy in reducing viral loads and alleviating symptoms during active infection. This study had an insufficient sample size to show the reduction in symptom severity with LC-Plasma; the number of participants should have been increased. Although it was difficult to show a reduction in symptom severity, LC-Plasma could reduce the length of symptom-positive days. This reduction in the number of days may have attenuated the burden on participants and provided a meaningful benefit. Despite these limitations, the findings support the potential of LC-Plasma supplementation as an accessible strategy to improve immune defence, especially for healthcare-associated populations and other high-risk populations.

## Figures and Tables

**Table 1:** WURSS-21 score for URI.

	Score	4	3	2	1
URI score WURSS21	Runny nose	None	Slight	Moderate	Severe
	Plugged nose	None	Slight	Moderate	Severe
	Sneezing	None	Slight	Moderate	Severe
	Sore throat	None	Slight	Moderate	Severe
	Scratchy throat	None	Slight	Moderate	Severe
	Cough	None	Slight	Moderate	Severe
	Hoarseness	None	Slight	Moderate	Severe
	Head congestion	None	Slight	Moderate	Severe
	Chest congestion	None	Slight	Moderate	Severe
	Feeling tired	None	Slight	Moderate	Severe

**Table 2:** Background information of participants at enrollment.

Parameters	LC-Plasma Group	Control Group
Body weight (kg)	55.0 ± 8.7	55.8 ± 8.6
Height (cm)	161.0 ± 8.0	162.3 ± 8.6
Gender(M/F)	18/34	26/23

**Table 3:** URI symptoms analysis (daily average score of WURSS 21 score).

Symptoms	LC-Plasma Group	Control Group
Runny nose	3.85 ± 0.20	3.85 ± 0.23
Plugged nose	3.87 ± 0.20	3.86 ± 0.24
Sneezing	3.89 ± 0.17	3.92 ± 0.16
Sore throat	3.93 ± 0.13	3.92 ± 0.12
Scratchy throat	3.94 ± 0.13	3.92 ± 0.14
Cough	3.90 ± 0.17	3.86 ± 0.21
Hoarseness	3.94 ± 0.12	3.95 ± 0.09
Head congestion	3.93 ± 0.12	3.93 ± 0.14
Chest congestion	3.98 ± 0.09	4.00 ± 0.02
Feeling tired	3.91 ± 0.13	3.84 ± 0.27
Total	39.16 ± 1.19	39.06 ± 1.04

**Table 4:** Cumulative fever days during the intervention period.

Groups	Fever Positive	Fever Negative	*p*-Value	Odds Ratio
LC-Plasma group	10	1446	0.029	0.442 (0.208–0.941)
Control group	20	1263		

**Table 5:** Cumulative Feeling-tired days during the intervention period.

Groups	Tired +	Tired −	*p*-Value	Odds Ratio
LC-Plasma group	99	1357	0.001	0.648 (0.507–0.828)
Control group	144	1228		

**Table 6:** Gastrointestinal infectious disease-related symptoms and general well-being score (daily average score).

Symptoms	LC-Plasma Group	Control Group
Diarrhea	3.99 ± 0.03	3.98 ± 0.08
Stomach pain	3.98 ± 0.04	3.94 ± 0.15
General well-being score	1.89 ± 0.80	2.07 ± 0.79

## Data Availability

All data supporting the conclusions were presented in the manuscript. Additional information will be made available by the corresponding author upon request.
